# ART and Serum albumin are influencing factors of the 5-year survival rate of people living with HIV undergoing maintenance hemodialysis caused by HIV: A cohort study

**DOI:** 10.1097/MD.0000000000035494

**Published:** 2023-10-06

**Authors:** Chunxiong Su, Yuting Ma, Huiping Liang, Aixian Huang, Wenhai Deng, Jia Zhou, Huaying Liu

**Affiliations:** a Department of Blood Purification, the Fourth People’s Hospital of Nanning, Guangxi (Guangxi AIDS Clinical Treatment Center), Nanning, China; b Department of Traditional Chinese Medicine, the Fourth People’s Hospital of Nanning, Guangxi (Guangxi AIDS Clinical Treatment Center), Nanning, China; c Department of Medicine, GuangXi Medical College, Nanning, China.

**Keywords:** antiretroviral therapy, human immunodeficiency virus, serum albumin, survival status

## Abstract

Human immunodeficiency virus (HIV) infection is one of the most prominent public health problems worldwide. The 5-year survival rate of people living with HIV undergoing maintenance hemodialysis (MHD) and the factors related to the survival rate have not been widely studied. This study calculated the 5-year survival rate of people living with HIV who were undergoing MHD and determined the risk factors that may affect the 5-year survival rate. All enrolled participants were followed up for more than 5 years from the first round of MHD. The survival rate of them was calculated, the Cox proportional hazards model was used for multivariate analysis, the Kaplan–Meier method was used to draw the survival curve, and the log-rank test was used to compare the survival time of different groups. A total of 121 participants were included in the study. Statistical analysis showed that the overall 5-year survival rate was 19.0%. The 6-, 12-, 24-, and 36-month survival rates were 71.90%, 56.20%, 41.32%, and 30.58%, respectively. Infection was the leading cause of death, accounting for 55.37%. The Cox proportional hazards model revealed that antiretroviral therapy (ART) and the serum albumin level after dialysis were independent protective factors for patient survival. The log-rank test showed that there was a significant difference in survival time between the ART and non-ART groups.

## 1. Introduction

Human immunodeficiency virus/acquired immune deficiency syndrome (HIV/AIDS) is a chronic infectious disease causing serious social harm. Since the first discovery of HIV-infected people in 1981, HIV/AIDS has become one of the most prominent global public health problems. At present, there are approximately more than 38 million HIV-infected people in the world, and more than 33 million lives have been lost.^[1–3]^ HIV epidemic monitoring data show that with rapid economic development, people’s living standards are rapidly improving, and their life expectancy is increasing.^[4]^ Prolonging the life of people living with HIV and improving their quality of life have always been the focus of research.

With the wide application of antiretroviral therapy (ART), the survival period of people living with HIV has been continuously extended, and the life expectancy of them is now close to that of ordinary people. HIV/AIDS has become a “chronic disease” that can be treated but not completely cured.^[5–7]^ With the continuous extension of the survival period of people living with HIV, they are facing increasing challenges. Previous studies have reported that after widespread use of ART, the prevalence of HIV-associated nephropathy (HIVAN) has been declining.^[8,9]^ However, HIVAN is still a research hotspot in recent years.^[10,11]^ HIVAN is a major complication in people living with HIV that can involve the glomeruli, renal tubules, renal interstitium, blood vessels and other body parts and is mainly caused by HIV-1 infection. Because the specific mechanism of HIVAN is not clear and no good treatment has been found, some of them will face end-stage renal disease and will consequently have to accept maintenance hemodialysis (MHD).^[12]^ For these people, it is worth discussing how to improve the dialysis effect, prolong their survival time and improve their quality of life.

However, related studies on the 5-year survival rate and the influencing factors for survival of these people are still rare. The purpose of this study was to determine the risk factors that may affect the 5-year survival time of people living with HIV on MHD and to provide a reference for the treatment of such people. In addition, we also wanted to use the research results as strong evidence to persuade people to receive ART as soon as possible.

## 2. Methods

### 2.1. Research object

People living with HIV who underwent MHD caused by HIV at Nanning Fourth People’s Hospital from June 2011 to May 2017 were selected as the participants. The inclusion criteria included the following: 18 years ≤ age ≤ 70 years; met the diagnostic criteria of HIV infection, and diagnosis was confirmed as HIV-1 infection by western blot test; met the diagnostic criteria of chronic kidney disease (CKD) stage 5 caused by HIV and met the indications of MHD; accepting the same ART scheme; and continuous telephone follow-up, participants should agree to receive a monthly telephone follow-up and cooperate in completing the relevant follow-up content. Exclusion criteria included patients requiring MHD due to acute renal failure; patients with acute myocardial infarction, severe respiratory failure and other serious respiratory and circulatory diseases; patients with hypertension, diabetes, nervous system diseases, liver dysfunction, serious digestive system diseases and other malignant tumor diseases; incomplete clinical data; those withdrawal from follow-up halfway participants were not part of the analysis; and deaths unrelated to HIV infection (such as drug overdose, accidents, suicide, and side effects of antiviral drugs). This study was approved by the medical ethics committee of Nanning Fourth People’s Hospital, and all enrolled subjects signed informed consent forms.

### 2.2. Data collection

The following data were collected: demographic data, baseline immunologic and virological characteristics, the time of diagnosis of HIV-1 infection, the antiviral treatment scheme, CD4^+^ T cells, CD8^+^ T cells, the CD4^+^/CD8^+^ ratio, renal function and other blood biochemical indicators, nutritional status, comorbidities, treatment plans, the start time of MHD, time of death and cause of death of people living with HIV. Regarding comorbidities, the diagnostic criteria for hypertension referred to the new ACC/AHA hypertension guidelines.^[13]^

### 2.3. Observation indicators

The starting point of follow-up observation was the time when the participants started MHD, death related to HIV infection was the outcome and the end point was after 5 years of follow-up. Participants with censoring events that referred to death unrelated to HIV-infection (including death caused by other diseases such as cardiovascular and cerebrovascular diseases or drug overdose, accidents, suicide, side effects of antiviral drugs and other nondisease causes) and loss of follow-up were not included in the statistical analysis.

### 2.4. Statistical processing

SPSS 21.0 software (IBM Corp., Armonk, NY) was used to analyze the data. Count data were expressed herein as n (%), and normally distributed continuous data were expressed as the mean ± standard deviation. The baseline demographic data and clinical and laboratory indicators of the study subjects were statistically described, and the differences in the secondary classification and count data were assessed using the χ^2^ test. A *t* test was used to analyze the difference in continuous normally distributed data. The measurement data of skewed distribution were expressed by the median (Percentiles 25, Percentiles 75), and Mann–Whitney *U* test was used for statistical analysis. Variables with *P* < .05 in univariate analysis were included in the Cox proportional hazards regression model for multivariate analysis, and the Kaplan–Meier method was used to plot the survival curve of people living with HIV undergoing MHD with and without antiviral treatment. *P* < .05 was considered to indicate statistical significance.

## 3. Results

### 3.1. General information

A total of 121 participants met the research criteria and were included in the statistical analysis, with an average age of 54.46 ± 12.67 years and an average body mass index (BMI) of 21.48 ± 4.05 kg/m^2^. Among them, 79.33% (96/121) were male, with an average age of 55.20 ± 12.51 years, 67.71% (65/96) received ART and the average BMI was 22.01 ± 4.16 kg/m^2^. Women accounted for 20.67% (25/121), with an average age of 51.64 ± 13.09 years; 68.0% (17/25) received ART and the average BMI was 19.41 ± 2.82 kg/m^2^. Other basic information is shown in Table 1.

**Table 1:** Summary of basic data of research participants.

Characteristics	Before MHD	After MHD
	Mean	Mean
CD8^+^ T lymphocytes, n/μL	538.07 (302.00, 694.00)	579.59 (318.00, 709.00)
CD4^+^ T lymphocytes, n/μL	239.01 (111.00, 344.00)	259.97 (118.00, 336.00)
CD4^+^/CD8^+^	0.53 (0.28, 0.75)	0.51 (0.23, 0.66)
Creatinine, μmol/L	884.57 (556, 1184.90)	571.13 (285.88, 706.75)
Creatinine clearance, mL/min	13.35 (3.39, 19.61)	20.42 (5.46, 21.61)
BUN, mmol/L	29.91 ± 15.13	25.84 ± 88.78
Blood potassium, mmol/L	4.20 ± 1.01	4.17 ± 0.98
Hemoglobin, g/L	76.44 ± 19.13	78.33 ± 16.97
ALB, g/L	33.36 ± 7.59	32.47 ± 6.99
PLT, ×10^9^/L	203.45 ± 101.78	180.41 ± 93.30
Cholesterol, mmol/L	7.55 ± 36.30	4.63 ± 3.18

ALB = albumin, BUN = blood urea nitrogen, PLT = platelet.

### 3.2. Five-year survival rate

**Figure 1.**
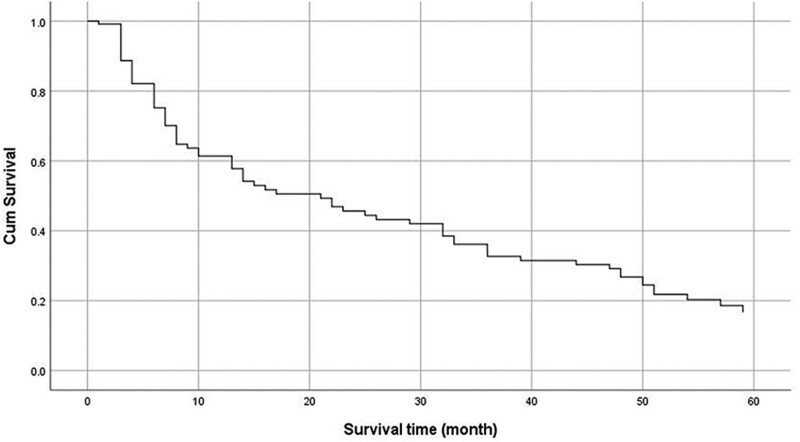
Five-year survival rate.

Among the 121 people who were followed up in this study, the overall 5-year survival rate was 19.0% (23/121) from the first dialysis. Among those still alive, the longest duration of dialysis was 126 months. The 6-, 12-, 24-, and 36-month survival rates were 71.90%, 56.20%, 41.32%, and 30.58%, respectively. As shown in Figure 1. Among our participants, 74/121 (61.16%) had tested for viral load. Among them, 62/83 (74.70%) of ART-participants received viral load testing, and the results showed that 41/62 (66.13%) had a test result of 0 copies/mL. Participants who did not receive ART had viral loads exceeding 10000 copies/mL.

### 3.3. Composition of the cause of death

Among all the participants who were followed up, a total of 98 HIV-related deaths occurred. According to the analysis of the causes of death, infection was the leading cause of death, accounting for 55.37% (67/121), followed by electrolyte disorder, accounting for 14.88% (18/121), heart failure, accounting for 9.09% (11/121), and multiple organ failure, accounting for 8.26% (10/121). In all cases of infection, lung infection accounted for 79.1% (53/67), which represents the most important cause of death, as shown in Table 2.

**Table 2:** Causes of death during the follow-up of people living with HIV on MHD.

Cause, n = 121	Infection	Renal failure	Metabolic disorder	Heart failure	Multiple organ failure	Others
N (%)	67 (55.37%)	25 (20.66%)	18 (14.88%)	11 (9.09%)	10 (8.26%)	12 (9.92%)

### 3.4. Cox proportional hazards regression model (univariate and multivariate analysis)

Univariate analysis was conducted with the 5-year survival as the dependent variable and other factors as the independent variables. The results showed that ART (63 (64.29%) vs 20 (86.95%), *P* = .045), blood urea nitrogen (BUN) after dialysis (17.84 ± 11.99 vs 60.05 ± 202.52, *P* = .44), serum albumin (ALB) level before dialysis (32.48 ± 6.86 vs 37.11 ± 9.39, *P* = .008), ALB level after dialysis (31.31 ± 6.25 vs 37.45 ± 7.93, *P* < .001) and the time from diagnosis to ART (16.00(1.00, 39.00) vs 36.00(12.00, 72.00), *P* = .024) were potential factors affecting the 5-year survival of people living with HIV undergoing MHD (see Table 3). The variables with *P* < .05 in univariate analysis were included in the multivariate Cox regression model. The results showed that ART (*P* = .009, OR = 0.529, 95% CI = 0.328–0.851) and ALB level after dialysis (*P* < .001, OR = 0.945, 95% CI = 0.916–0.975) were independent protective factors for 5-year survival of people living with HIV undergoing MHD (see Table 4).

**Table 3:** Univariate analysis of predictors for 5-year survival.

Characteristics	More than 5-year survival group, N = 23	Less than 5-year survival group, N = 98	*P*
Sex (F/M)	5/18	20/78	.887
Age, yr	53.96 ± 12.52	54.58 ± 12.75	.832
BMI, kg/m^2^	22.01 ± 4.38	21.34 ± 3.98	.482
ART, n (%)	20 (86.96%)	63 (64.29%)	.045
Anemia, n (%)	23 (100%)	92 (93.88%)	.266
Characteristics before MHD			
CD8^+^ T lymphocytes, n/μL	407.00 (302.00, 729.25)	459.00 (302.00, 684.00)	.637
CD4^+^ T lymphocytes, n/μL	246.00 (142.50, 350.00)	202.00 (96.50, 333.50)	.316
CD4^+^/CD8^+^	0.45 (0.30, 0.84)	0.44 (0.28, 0.75)	.539
Creatinine, μmol/L	714.60 (508.80, 1283.00)	764.55 (564.60, 1164.73)	.819
Creatinine clearance, mL/min	12.82 (5.54, 19.33)	7.79 (2.61, 19.63)	.136
BUN, mmol/L	28.41 ± 13.56	30.25 ± 15.51	.609
Blood potassium, mmol/L	4.29 ± 1.32	4.18 ± 0.93	.644
Hemoglobin, g/L	78.28 ± 24.07	76.00 ± 17.89	.609
ALB, g/L	37.11 ± 9.39	32.48 ± 6.86	.008
PLT, ×10^9^/L	209.26 ± 112.77	202.07 ± 99.58	.762
Cholesterol, mmol/L	3.98 ± 1.10	8.30 ± 39.93	.631
Characteristics after MHD			
CD8^+^ T lymphocytes, n/μL	709.00 (254.00, 1243.00)	490.00 (319.50, 632.25)	.082
CD4^+^ T lymphocytes, n/μL	238.00 (120.00, 574.00)	233.50 (111.25, 318.50)	.344
CD4^+^/CD8^+^	0.35 (0.25, 0.47)	0.46 (0.23, 0.79)	.171
Creatinine, μmol/L	420.30 (282.48, 567.48)	495.70 (287.03, 715.00)	.361
Creatinine clearance, mL/min	19.01 (10.54, 25.34)	9.45 (4.51, 16.61)	.12
BUN, mmol/L	60.05 ± 202.52	17.84 ± 11.99	.044
Blood potassium, mmol/L	4.29 ± 1.32	4.18 ± 0.93	.109
Hemoglobin, g/L	84.75 ± 19.84	76.79 ± 15.95	.048
ALB, g/L	37.45 ± 7.93	31.31 ± 6.25	.000
PLT, ×10^9^/L	193.77 ± 92.23	177.22 ± 93.77	.457
Cholesterol, mmol/L	4.68 ± 1.25	4.62 ± 3.45	.948
Time from diagnosis to ART, mo	36.00 (12.00, 72.00)	16.00 (1.00, 39.00)	.024

ALB = albumin, ART = antiretroviral therapy, BMI = body mass index, BUN = blood urea nitrogen, PLT = platelet.

**Table 4:** Multivariate Cox regression model on survival time of people living with HIV undergoing MHD.

Characteristics	Wald	Sig.	OR	95% CI for Exp(B)	
				Lower	Upper
ART	6.873	0.009	0.529	0.328	0.851
ALB after dialysis	12.838	0.000	0.945	0.916	0.975

ALB = albumin, ART = antiretroviral therapy, MHD = maintenance hemodialysis.

### 3.5. Survival curves of 2 groups receiving antiviral treatment and not receiving antiviral treatment

**Figure 2.**
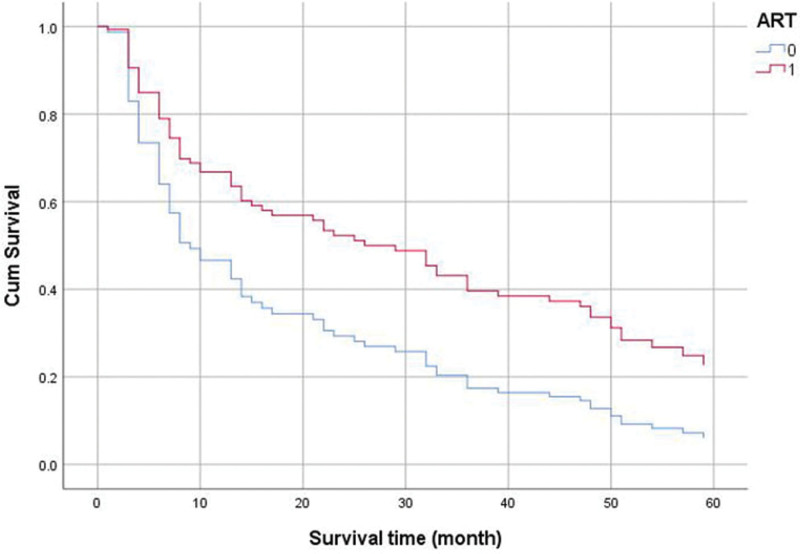
Survival curves of 2 groups receiving ART and not receiving ART. ART = antiretroviral therapy.

The Kaplan–Meier method was used to plot the survival curve of people living with HIV undergoing MHD with and without ART. The results are shown in Figure 2. The log-rank (Mantel Cox) test revealed that ART significantly prolonged the survival time (χ^2^ = 11.747, *P* = .001).

## 4. Discussion

Our study was aimed to explore the 5-year survival rate of people living with HIV undergoing MHD and determine the factors affecting the 5-year survival rate. The results showed that the overall 5-year survival rate of the these people was 19.0% (23/121). The 6-, 12-, 24-, and 36-month survival rates were 71.90%, 56.20%, 41.32%, and 30.58%, respectively. Infection was the leading cause of death. The ALB levels after dialysis and ART were independent protective factors for patient survival.

With the advent of the ART era, an increasing number of people living with HIV can achieve long-term and prolonged survival. However, in the process of coexisting with the virus for a long time, with the extension of the infection time, the risk of opportunistic infection continues to rise, and various organs, such as the kidneys, of many patients will be damaged.^[14]^ The renal function damage caused by HIV infection, including abnormal kidney morphology, physiology, pathology and function, is often related to the course of HIV infection course, virus load status and drug treatment, infection, tumorigenesis and metabolic disorders.^[15]^ The specific mechanism of HIV/AIDS combined with renal function damage is not completely clear at present. However, the reasons may be as follows: Firstly, HIV directly invades the kidney and causes renal damage and immune dysfunction caused by HIV infection leads to opportunistic infection in people living with HIV. Second, the actions of inflammatory factors, immune injury and metabolic disorders impact renal function. Third, people are experiencing adverse effects of ART drugs and other factors. Finally, people may have combined basic diseases and complications such as hypertension, diabetes, chronic hepatitis B/C and cardiopulmonary dysfunction.^[16,17]^ With the survival time of people living with HIV increasing, the influencing factors and prognosis in such cases have attracted increasing attention. In previous clinical studies, the mortality rate of people living with HIV on MHD was high. For example, Trullàs et al^[18]^ reported that the medium-term survival rate of people living with HIV receiving MHD treatment was significantly lower than that of controls. The results showed that the overall 5-year survival rate was 19.0%, and the 6-, 12-, 24-, and 36-month survival rates were 71.90%, 56.20%, 41.32%, and 30.58%, respectively. This is similar to the results of Atta et al.^[19]^ Although their study was conducted in the USA, with geographic or racial differences, they also reported that among those patients who initiated renal replacement therapy during the ART era, 87 deaths occurred (73%). The researchers believed that the survival rate of those people living with HIV with end-stage renal failure who receive renal dialysis is not high because of the ART. Therefore, we believe that for people with HIVAN, the 5-year survival rate is not high enough when they need MHD. It is of great guiding significance to study the factors influencing death and the prognosis of people living with HIV undergoing MHD in order to prolong the survival of such people.

We further examined statistics on the causes of death of people living with HIV undergoing MHD. Among the HIV-related causes of death, the first cause of death affecting the 5-year survival rate was infection (55.37%), the second was electrolyte disorder (14.88%), the third was heart failure (9.09%), and the fourth was multiple organ failure (8.26%). In all cases of infection, lung infection accounted for 79.1% (53/67), which represents the most important cause of death in such people. However, the main causes of death in MHD patients in the general population are cardiovascular disease and infection.^[20,21]^ These results are basically consistent with those reported by Tourret et al^[22]^ Community-acquired pneumonia is the most common cause of hospitalization for people living with HIV and is the main cause of death.^[23,24]^ Since the introduction of antiviral treatment, in high-income countries, opportunistic respiratory infections such as Pneumocystis pneumonia, are no longer the main reason for people living with HIV to be admitted to the ICU. However, in developing countries, respiratory failure caused by Pneumocystis pneumonia is still relatively common.^[25]^ Due to the lack of immunity of people living with HIV, the risk of various opportunistic infections increases. Once infection occurs, the people’s immune system cannot work normally, which easily causes serious bacteremia, toxemia, and even multiple organ failure.

We further conducted univariate analysis on the factors affecting the 5-year survival rate of people living with HIV undergoing MHD. According to whether the participant survived for more than 5 years, they were divided into 2 groups. Univariate analysis was conducted with the 5-year survival period as the dependent variable and other factors as the independent variables. The results showed that ART, BUN levels after dialysis, ALB levels before dialysis, ALB level after dialysis and the time from diagnosis to ART were potential factors affecting the 5-year survival rate of people living with HIV undergoing MHD. The variables with *P* < .05 in the univariate analysis were included in the multivariate Cox regression model. The results showed that ART was an independent protective factor for the survival of people living with HIV undergoing MHD. This is consistent with many previous studies. In prior studies, ART was considered an independent protective factor against death in people living with HIV. ART is currently the most effective treatment for HIV infection.^[26]^ Antiviral therapy can maximally suppress the viral load for a long time, rebuild and maintain the immune function of the body, and reduce the morbidity and mortality of human immunodeficiency virus-related diseases. Moreover, such therapy can effectively reduce the HIV viral load in people living with HIV, delay the infection process, help the body carry out immune reconstruction, reduce the risk of mother-to-child transmission, and improve the survival rate and quality of life.^[27]^ ART can rebuild the immune system of the body by regulating the number of T lymphocytes, the quality of CD4^+^ T lymphocytes and the activation status of T lymphocytes to achieve anti-HIV infection and disease control. ART can effectively increase the number of T lymphocytes, strengthen the antiviral effects of the immune system, significantly reduce the viral load and increase the number of peripheral CD4^+^ T lymphocytes. This effect is mainly related to the expansion of cell clones, the redistribution of retained memory T lymphocytes and the reduction in apoptotic cells. After 2 to 3 months of ART treatment, the number of CD4^+^ T lymphocytes will increase steadily and slowly, lasting for more than 1 year. The increased CD4^+^ T lymphocytes at this stage are mainly I cells. This is mainly because after effectively inhibiting the virus, the thymus can express new CD4^+^ T lymphoid cells, thus increasing the number of periphInaive cells.^[28–30]^ In addition, during ART treatment, the number of CD8^+^ T lymphocytes will also increase significantly, with rapid growth in the initial stage and slow growth in the later stage. After ART treatment, the specific antigen response ability of memory CD4^+^ T lymphocytes in people living with HIV can be effectively restored, and the humoral immune response will gradually recover. A previous study showed that after ART, the immune activation status was significantly reduced, the levels of various activation markers were significantly decreased, and the levels of chemokine receptor 4 on the cell surface were significantly increased.^[31]^ Therefore, we also support the use of ART for people living with HIV who meet the application standards as much as possible, as this can significantly prolong their survival time. We used the Kaplan–Meier method to plot the survival curve of people living with HIV undergoing MHD with and without antiviral treatment. The results showed that the survival curve of those people receiving ART was prolonged, and the log-rank test suggested that ART could significantly prolong their survival time (χ^2^ = 11.747, *P* = .001).

Our study also revealed that the ALB level after dialysis of people living with HIV undergoing MHD was an independent protective factor for patient survival. At present, ALB, as an index to measure the nutritional status and inflammation of patients, has been confirmed by many clinical studies to be significantly related to the prognosis of MHD. Atta et al^[19]^ found that among those people living with HIV, a low ALB level was a risk factor for death. A low nutritional level will have a great impact on the autoimmune function, which will not only cause great damage to the patient’s physiological function but can also lead to a significant decline in the patient’s quality of life and ability to perform daily activities.^[32]^ In addition, research data also showed that the average resting energy consumption of the body tissues of people living with HIV was large, while they had different degrees of nutrient absorption barriers, and the energy consumed was far greater than the energy absorbed by the body, which promoted the decline in resistance and became the main cause of tumor diseases and infectious diseases. Moreover, relevant research has shown that the nutritional status of people living with HIV directly affects their disease control ability and survival rate. Improving the nutritional status can not only improve the clinical treatment effect but can also effectively enhance their immunity, which plays an important role in alleviating clinical symptoms.^[33–35]^ We believe that for those people living with HIV undergoing MHD, it is necessary to monitor their nutritional status, especially the ALB level after dialysis, to carry out targeted nutritional support treatment in a timely manner for malnourished people, thereby effectively ensuring the effect of AIDS treatment, prolonging the survival time of these individuals and improving their quality of life.

## 5. Study limitations

Nevertheless, our study has certain limitations. First, the survival rate is affected by many factors. Some clinically unknown factors may not be included in the statistical considerations of this study. Second, the present study mainly included participants who had been followed up for more than 5 years. The number of cases is thus relatively small. The more cases observed, the higher is the reliability of the data, and the more scientific the clinical management guidance provided. Third, during the follow-up process, there was always some bias. Fourth, the types of kidney diseases may influence the prognosis of people living with HIV undergoing MHD. However, due to economic reasons and concerns about complications, our participants did not undergo kidney biopsy to clarify the pathological type. Thus, we will continue to follow up and expand the number of study cases, include the types of kidney diseases in the research analysis in the future.

## Acknowledgments

We would like to thank Mr. Zhizhong Li who is a professor of statistical for collating and analyzing clinical data.

## Author contributions

**Conceptualization:** Chunxiong Su, Yuting Ma, Huaying Liu.

**Data curation:** Chunxiong Su, Aixian Huang, Wenhai Deng, Jia Zhou.

**Formal analysis:** Huiping Liang, Huaying Liu.

**Investigation:** Aixian Huang, Wenhai Deng, Jia Zhou.

**Methodology:** Chunxiong Su, Yuting Ma, Huiping Liang, Huaying Liu.

**Project administration:** Chunxiong Su, Huaying Liu.

**Supervision:** Chunxiong Su, Yuting Ma, Huaying Liu.

**Writing – original draft:** Chunxiong Su, Yuting Ma, Huaying Liu.

**Writing – review & editing:** Huaying Liu.
